# Saudi Health System and Health Security Structure: A Scope Review Study Addressing the National Need for Governing the Health Security

**DOI:** 10.7759/cureus.47376

**Published:** 2023-10-20

**Authors:** Hani S Almugti, Amal A Aldeghalbey, Khadijah A Swaif, Hind H Alrashdi, Estabraq M Mahdi, Maram B Alharbi, Abrar S Alsaidi, Norah Y Algathradi, Salwa M Alanazi, Najeeb S Alsalameh, Atyaf Kariri, Enas A Alasmari, Khalid A Alqarni, Ebtehal J Asiri, Jumana H Alhasan

**Affiliations:** 1 Primary Health Care, Ministry of National Guard - Health Affairs, King Abdullah International Medical Research Center, King Saud bin Abdul-Aziz University for Health Sciences, Jeddah, SAU; 2 Pharmacy, King Fahad Military Medical Complex, Jeddah, SAU; 3 Prehospitalisation Department, Hospital Dhahran Eye Specialist Hospital, Dhahran, SAU; 4 Molecular Biology Department, Jeddah Regional Laboratory, Jeddah, SAU; 5 Respiratory Therapy, King Abdullah Medical City, Makkah, SAU; 6 Pharmacy, Dr.Suliman Alhabib Medical Group, Riyadh, SAU; 7 Laboratory Medicine, Jeddah Regional Laboratory, Jeddah, SAU; 8 Internal Medicine, Khamis Mushait General Hospital, Khamis Mushait, SAU; 9 Family Medicine, Aldefea Primary Health Care Center, Al-Madinah al-Munawwarah, SAU; 10 Psychiatry, Iradah Mental Complex, Buraydah, SAU; 11 Medicine, College of Medicine, Jazan University, Jazan, SAU; 12 Research and Studies Department, Directorate of Health Affairs, Jeddah, SAU; 13 Emergency Department, Thuryban General Hospital, Al Qunfudhah, SAU; 14 Public Health, King Khalid University, Abha, SAU; 15 Medicine, Fakeeh College of Medical Sciences, Jeddah, SAU

**Keywords:** saudi arabia, global health security, public health, health system, health security

## Abstract

Health security has gained significant attention at the national and global levels, “security” is not a simple term; instead, it is “essentially contested” - that is, it induces debates about what it means and how to use it. This study aimed to define three terms frequently used in health security discussions. These terms are national health security, Global Health Security, and public health. The research method was a scoping review performed in three stages. The first stage was electronic searching based on selection criteria among multiple sources at various time points during the year 2023. These sources included online literature searches, websites of non-governmental organizations (NGOs), and other governmental health agencies. The second stage involved determining the relevance of the selected papers to the study’s objectives; the selected papers had moderate to high relevance to the study’s objectives. The third stage was to evaluate the methodological quality of a study; we selected peer-reviewed published papers and websites recognized as trustworthy sources of information. The search yielded 143 articles; five met the inclusion criteria and were subjected to the definition of health security. Despite proposed definitions, agreement has yet to be reached on the content and scope of health security. Another main finding is that health security requires more state and international collaboration efforts to reach Global Health Security. To the best of our knowledge, no known government body or organization is responsible for governing health security in Saudi Arabia. However, the current study presents a definition of health security and differentiates it from the public health approach, in addition to emphasizing the importance of governing the related health sectors within each country in order to improve health security and have a positive impact on overall Global Health Security.

## Introduction and background

One of the top priorities of the Saudi government is the development of its healthcare system, which has been recognized as one of the most advanced in the Middle East [1]. In Saudi Arabia, various public and private healthcare facilities, including hospitals and clinics, are supervised by the Ministry of Health (MOH), the primary government body responsible for providing healthcare [2]. In 2016, the Saudi government announced a strategic framework of initiatives to diversify Saudi Arabia’s economy and reduce its dependency on oil. These initiatives cover developing public service sectors, including health, education, infrastructure, recreation, and tourism [3]. As part of the Vision 2030 initiative, the Saudi Health Sector Transformation program was launched in 2021 to enhance healthcare services’ accessibility and quality through a comprehensive, efficient, and integrated health system [2,3].

The recent COVID-19 epidemic tested the capacity and readiness of healthcare systems worldwide. The high demand for hospitalization, critical care, and other medical procedures left many countries needing support to cope with these challenges. [4]. However, efficient management of the COVID-19 crisis through timely testing, contact tracing, and patient treatment has been mostly observed in countries with more resilient healthcare systems [5].

The government of Saudi Arabia had outstanding achievements by conducting a proactive and comprehensive approach to managing the COVID-19 pandemic. The MOH has been leading the country’s response, working closely with other governmental sectors and healthcare providers to implement various measures to control the spread of the virus [6]. Despite being highly advanced, healthcare systems worldwide have encountered various challenges during the COVID-19 pandemic, such as shortages of medical supplies and burnout among healthcare workers due to prolonged and intense workloads. Several studies conducted during the pandemic have highlighted the need for continued investment in healthcare systems to improve their capacity to respond to the next public health emergencies and ensure effective collaboration and communication between healthcare facilities and government agencies [7].

The term “health security” has recently gained significant attention at the national and global levels. Various studies have been conducted to support its use from health, national security, and political perspectives [8]. This study aimed to define health security within the context of the threats and risks in Saudi Arabia, as well as the political and administrative framework that governs the MOH with other relevant government entities.

## Review

Search strategy and selection criteria

The review was conducted using the following electronic databases: PubMed, MEDLINE, EMBASE, Cochrane Library, International Health Regulations (IHRs), and World Health Organization (WHO) Global Database. The electronic search for articles and papers using keywords and phrases provided the foundation for searching. It was derived from the review objectives (“public health security” OR “health security” OR “Saudi health security” OR “Global health threats*”).

Criteria for inclusion and exclusion studies

This review considered the following studies: English language studies; published and peer-reviewed articles of the last 20 years; studies that discussed the definition of health security and the key variations in how the concept was interpreted and applied in medical field contexts; studies that discussed the major public health events in Saudi Arabia from a historical point of view; previous reviews were included since the present review aimed to identify key terms and trends rather than extracting data for meta-analysis; individual studies included in meta-analyses were included.

Publication selection and method of the review

The researchers of the present study were enlisted into three groups: The first group was assigned to an online search based on inclusion criteria; their results were imported into Excel. The second group was assigned to screening the title and abstract and categorizing the articles into high and/or medium relevance according to the research objectives of the present study as the following: (High: The publication makes direct links with research objectives of the present study; Medium: The publication presents general and not specific link with research objectives of the present study; Low: The publication presents low links with research objectives of the present study; however, articles with low relevance were excluded). The third group was assigned to incorporate a process of critique or appraisal of the evidence. This appraisal aimed to assess a study’s methodological quality and determine how much it had addressed the possibility of bias in its design, conduct, and analysis. Based on the voting system and after assessing the quality of each article, the investigators in this group decided to include or exclude the selected article. However, the appropriate (The Joanna Briggs Institute) JBI critical appraisal tool was used based on the included study design. It is available on this website: https://joannabriggs.org/critical-appraisaltools.

Result and findings

**Figure 1:**
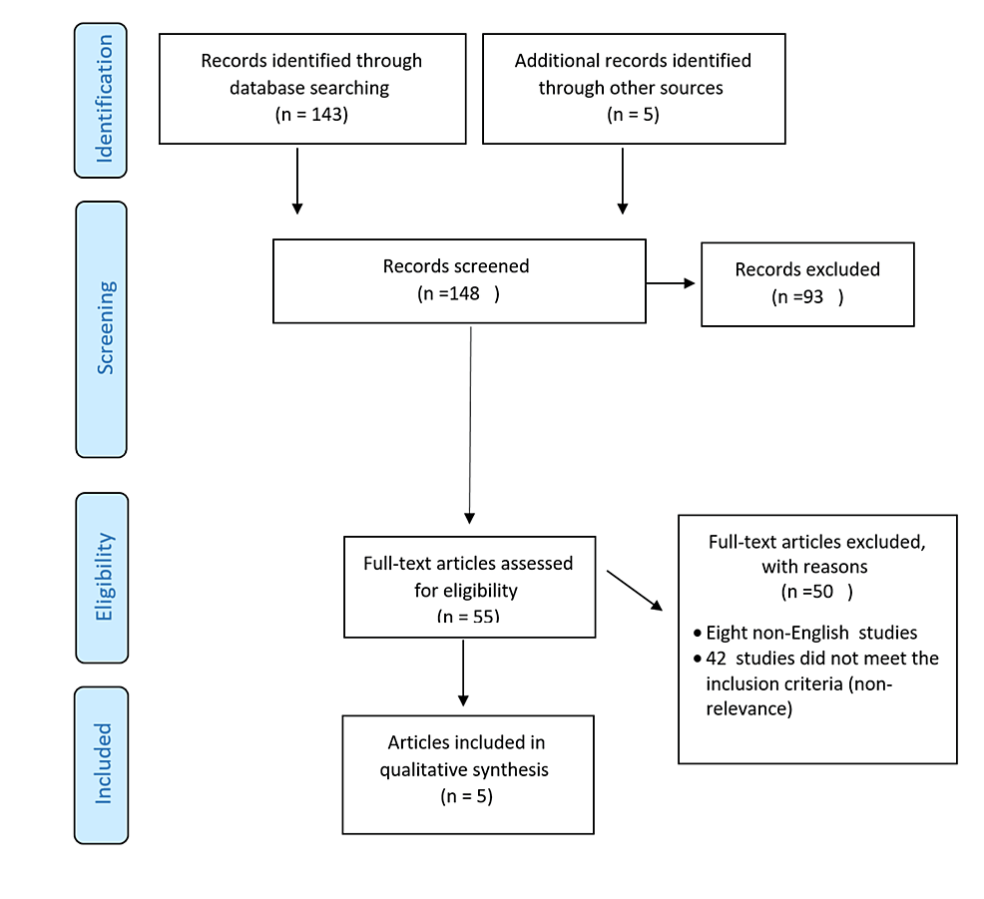
Flow diagram of study selection procedures This flow diagram was prepared by authors of this article.

As summarized in Figure 1, the search yielded 143 articles; five met the inclusion criteria and were subjected to the health security definition.

Data were gathered from multiple sources at various time points during the year 2023, and these sources were online literature searches, non-governmental organization (NGO), and agency websites as the following:

Medical Literature

A literature review identified several studies that generally discussed health security and its association with other health aspects instead of clearly defining the scope and content of this term. For instance, some studies linked health security to patient safety, while others discussed it from human rights and political perspectives [8-11]. Moreover, a few studies discussed health security in the context of communicable diseases and highlighted the importance of international cooperation in preventing them [4,12]. From the Saudi medical literature, no study addressing health security has been published. However, the publication selection and review methodology criteria were met in the following studies below in Table 1.

**Table 1:** Characteristics of included studies

Authors	Title	Study Setting	Study design	Publication Date	Study quality
Aldis [8]	Health security as a public health concept: A critical analysis	Online literature searches	Literature review	2008	Good
Augustynowicz et al. [13]	Health security: Definition problems	Poland literature	Scoping review	2022	Good
Šehović [14]	Towards a new definition of health security: A three-part rationale for the twenty-first century	Online literature searches	Literature review	2020	Good
Lentzos et al. [15]	Health security intelligence: Engaging across disciplines and sectors	Online literature searches	Literature review	2020	Good
Rigby [16]	Redefining security – lessons from public health	Group discussion	Commentary	2015	Good

The first study addressed the intersection of health security with public health and the role of the “health security” concept in current public health practice [8]. This study provides some differences between public health and health security. Public health has a primary healthcare approach, emphasizing community involvement, and protection of vulnerable groups. On the other hand, health security is located at the national level, protecting national populations against external threats such as bioterrorism, pandemics, and other major threats. This study concluded that although the term is becoming more widely accepted in the literature of public health practice, there has yet to be a consensus on the scope or content of health security.

The second study is a scoping review from Poland in 2022, conducted to find a suitable definition of health security [13]. In this study, they considered the provision of healthcare access as a health security factor to achieve a high level of healthcare security. Furthermore, other health determinants, including social, economic, and environmental, necessitate a coordinated collaboration between the public and private sectors in order to attain healthcare security. Overall, this study has concluded that a precise definition of healthcare security needs to be improved, and it should be a comprehensive concept requiring further studies.

Lastly, despite the numerous health benefits of the current international health security strategies between countries, two studies underscored the need to improve collaboration among institutions of each country [14-15]. However, these studies in addition to commentary have consistently shown that the current health security definition of the WHO is insufficient without addressing the collaboration between government and non-government institutions within each country to strengthen GHS and effectively prevent, identify, and respond to health security threats [16].

Governmental and Nongovernmental Organizations (NGOs)

Health security has gained significant attention in recent years, particularly with the emergence of global health threats such as pandemics and bioterrorism. In 1994, The United Nations Development Programme (UNDP) report was among the first to introduce the term health security, incorporating it into the list of dimensions of human security based on human security threats that include economic, food scarcity, health, environment, personal, community, and political threats [17].

In 2003, the Commission on Human Security described GHS internationally as complementary to public security [18]. This commission reported to the Secretary General of the United Nations numerous recommendations, including a recommendation related to maintaining health security by enabling access to healthcare. Since then, health security has been recognized internationally as a critical component of public security, human rights, and human development.

The expansion of international travel and trade and the emergence or reemergence of infectious diseases and other public health hazards served as a warning signal for many countries to prepare and invest in healthcare systems with the urgent need for a global response against health threats. To address this, the WHO developed the International Health Regulations (IHR) in order to prevent, protect against, control, and respond to the international spread of diseases. In 2005, the IHR was revised to incorporate the concept of health security, which encompasses the control measures to minimize the risk and mitigate the impact of acute public health events that threaten people’s health across geographical regions and international borders [19].

It is worth mentioning that when the WHO issued its annual report in 2007, it adopted the definition of GHS as “the activities required, both proactive and reactive, to minimize vulnerability to acute public health events that endanger the collective health of populations living across geographical regions and international boundaries” [20]. This definition emphasizes the need for countries to take proactive measures to prevent and prepare for health threats rather than merely responding to outbreaks when they occur. It also recognizes the interconnectedness of health security across borders and the importance of global coordination in preventing and responding to health emergencies.

However, some countries worldwide are developing their strategies for healthcare security, including defining the operational definition of healthcare security and what it entails. For example, the recent US national strategy 2023 defines healthcare security as “a whole-of-nation approach to prepare for, protect from, respond to, and recover from the adverse health effects of public health emergencies and disasters” [21]. In other countries, such as the United Kingdom, The UK Health Security Agency (UKHSA) has been established and is responsible for health security by protecting every member of every community from the impact of biological, chemical, radiological, and nuclear incidents, and other health threats [22]. This agency provides intellectual, scientific, and operational leadership at the national and local levels and on the global stage to secure the nation’s health. In Australia, the definition of healthcare security is outlined in the Health Security Action Plan 2019-2023 as follows: “Health security requires limiting disorder and death cases associated with public health-related events by providing flexible and consistent systems that can be adapted and developing according to changes in diseases, societies, technologies, and information” [23].

Discussion

This scoping review study set out with the aim of defining health security. A common finding from reviewing the online resources is that health security requires more collaboration efforts at the state level and international levels to reach GHS. To quantify the country’s ability to prevent and mitigate any health threats, in 2019, the GHS Index was developed by a group of experts from the Johns Hopkins Center for Health Security, the Nuclear Threat Initiative (NTI), and the Economist Intelligence Unit (EIU) [24]. The GHS Index aims to inform decision-makers from each country about the fundamental components needed to prepare their nations for upcoming outbreaks and to help them decide where to focus their planning efforts and allocate long-term resources.

At the national level, another important finding is the necessity of collaboration among different government and private sectors to ensure health security inside each country. However, despite efforts to govern health security, the insufficient definition of health security poses a challenge. The combination of political or security terms with the health word introduces a conflict point about who will lead the health security efforts in a country. This aligns with the findings of a previous study that declared no consensus on the content and scope of national health security work [8].

As stated earlier, every country should strive to improve its healthcare system and public health programs in order to detect, prevent, and respond to health threats. This is consistent with previous research, which indicated that improving the healthcare system’s resilience is the key to protecting the health and enabling the system to assess the potential threat, based on which national preparedness is established and proposed mitigation action.

To the best of our knowledge, no known government body or organization is responsible for governing health security in Saudi Arabia. According to the GHS Index, health security in Saudi Arabia was ranked 62 out of 195 countries compared to the United States, which ranked first. This unexpected finding was discussed in a previous study [25]. This index did not reflect the government’s efforts to ensure national health security during past and present health crises, such as infectious diseases, terrorist attacks, and hostile military. It is difficult to explain this, but it might be related to the methodology of scoring data or the degree of availability of national data from national entities.

Finally, several important limitations need to be considered. First, because the studies with the English language from online resources were the only ones included in this present scoping review, there is an implicit assumption that results from previously unpublished or non-English studies should have been included. Second, the definition of health security threats was not presented in this study; however, addressing it would have helped to provide a more comprehensive understanding of the health security definition. Finally, a number of important limitations need to be considered. First, because the studies with the English language from online resources were the only ones included in this present scoping review, there is an implicit assumption that results from previously unpublished or non-English studies should have been included. Second, the definition of health security threats was not presented in this study; however, addressing it would have helped to provide a more comprehensive understanding of the health security definition.

## Conclusions

The present study was designed to define health security, to our knowledge, this is the first Saudi attempt to synthesize data from large and diverse bodies of international literature looking at the connections between public health and global and national health security. The investigators of the current study proposed a definition for health security as *a comprehensive national system encompassing all preparedness, response, and recovery activities that the state should lead to ensure readiness and effectiveness in protecting the population within its borders from all threats and incidents that pose risks to public health, whether internally, across borders, or internationally.* The study confirmed that despite the increasing recognition of health security in public health literature and practice, its scope or content needs to be clarified. Further, one of the more significant findings from this study is that enhancing the health security inside each country by governing the related sectors will positively reflect GHS.
